# Pericallosal artery aneurysms: an evidence-based analysis of clinical presentations, therapeutic approaches, and outcome

**DOI:** 10.1007/s10143-025-03500-6

**Published:** 2025-04-03

**Authors:** Amr Badary, Khadeja Alrefaie, Mohammed A. Azab, Yasser F. Almealay, Mohammed Q. Alibraheemi, Wireko Andrew Awuah, Alan Hernández-Hernández, Sura N. Alrubaye, Nabiha Midhat Ansari, Levent Tanrikulu, Oday Atallah

**Affiliations:** 1 Department of Neurosurgery, SRH Wald-Klinikum Gera, Academic Hospital of Jena University, Gera, Germany; 2 Faculty of Medicine, Royal College of Surgeons in Ireland, Busaiteen, Bahrain; 3 Departemnt of Neurosurgery, Cairo University Hospital, Cairo, Egypt; 4 Faculty of Medicine, University of Kufa, Kufa, Iraq; 5 Faculty of Medicine, Carol Davila University of Medicine, Bucharest, Romania; 6 Department of Research, Inter-Continental Omni-Research in Medicine Collaborative, Berlin, Germany; 7 Department of Neurosurgery, National Institute of Neurology and Neurosurgery, Mexico City, Mexico; 8 Faculty of Medicine, University of Babylon, Hilla, Iraq; 9 Faculty of Medicine, Medical University of Plovaffiliation, Plovaffiliation, Bulgaria; 10 Departemnt of Neurosurgery, Carl Von Ossietzky University Oldenburg, Oldenburg, Germany; 11 Department of Neurosurgery, Evangelic Hospital Oldenburg, Carl Von Ossietzky University Oldenburg, Oldenburg, Germany

**Keywords:** Pericallosal artery, Aneurysm, Distal anterior cerebral artery

## Abstract

Pericallosal artery aneurysms (PCAA) are relatively rare intracranial aneurysms that present unique challenges in diagnosis and management. This study provides a comprehensive review of the literature to assess demographic patterns, risk factors, treatment approaches, and complications associated with PCAA. Data from 23 studies were analyzed using Python with libraries such as Pandas and Matplotlib. Descriptive statistics and crosstabulations explored the relationships between treatment modalities (microsurgical, endovascular, combined) and complications, including hydrocephalus, vasospasms, and intraprocedural ruptures. Visualizations were employed to depict the prevalence and impact of various outcomes. Analysis revealed a notable gender disparity, with females constituting 70.47% of the study population. The average age was 49.93 years, and the average aneurysm size was 6.34 mm. A majority of aneurysms were ruptured (542 ruptured vs. 251 unruptured). Risk factors like smoking were prevalent, and radiological features such as subarachnoid hemorrhage (SAH) were commonly reported. Endovascular treatment was slightly more frequent (86.96%) than microsurgical treatment (73.91%). Vasospasm was the most reported complication (56.5%), followed by hydrocephalus and intraprocedural rupture. The analysis of the distribution of studies reporting complication for each treatment modality showed that endovascular treatment studies reported higher rates of vasospasms, hydrocephalus and intraprocedural rupture. The mortality rate was 6.52%, with a mean follow-up duration of 20.77 months. This review reveals that PCAAs predominantly affect females, with an average patient age of 49.93 years. Aneurysms averaged 6.34 mm and often caused SAH. Endovascular treatments were more common but had higher complication rates than microsurgical methods, which also carried risks. The mortality rate was 6.52%.

**Supplementary Information:**

The online version contains supplementary material available at 10.1007/s10143-025-03500-6.

## Clinical trial number

Not applicable.

**Supplementary Information:**

The online version contains supplementary material available at 10.1007/s10143-025-03500-6.

## Introduction

Pericallosal artery aneurysms (PCAAs) are relatively rare among cerebral aneurysms, accounting for less than 9% of all intracranial aneurysms and about 4% of ruptured intracranial aneurysms [1–3]. Despite their low prevalence, these aneurysms present unique challenges due to their complex anatomical location and the high risk of rupture [3]. The pericallosal artery, which supplies the corpus callosum and adjacent brain structures, is susceptible to aneurysm formation, often presenting a diagnostic and therapeutic challenge [4–6].

The pathogenesis of PCAAs can be congenital, although they can also be associated with vascular anomalies, trauma, or infections [3]. Hormonal factors, particularly estrogen, have been implicated in the higher incidence of these aneurysms among females and individuals between 40 and 60 years of age [7–12]. Furthermore, the presence of multiple aneurysms in a single patient has been reported in various studies, emphasizing the need for comprehensive risk assessment and management strategies [13, 14].

Clinical presentation of PCAA varies widely, with some cases being asymptomatic while others present with severe symptoms such as subarachnoid hemorrhage (SAH) or focal neurological deficits [15–17]. Accurate diagnosis often requires advanced imaging techniques, including computer tomography (CT) scans, magnetic resonance imaging (MRI)s, and cerebral angiography, to differentiate PCAAs from other types of intracranial lesions [18]. The management of PCAAs involves a range of treatment modalities, from traditional microsurgical clipping to modern endovascular techniques [19–22].Each treatment option carries specific risks and benefits, and the choice of approach can significantly impact patient outcomes [23, 24].

Complications associated with PCAAs include vasospasm, hydrocephalus, and intraprocedural rupture, which can influence overall treatment success and patient recovery [25, 26]. Vasospasm, for instance, is a common complication that may arise post-treatment and affect long-term prognosis [25]. Hydrocephalus, often resulting from SAH or surgical intervention, also poses a significant challenge in the management of these aneurysms [26].

This systematic review aims to synthesize current knowledge on PCAAs by examining epidemiological data, risk factors, treatment modalities, and associated complications [8–10, 27]. Through a comprehensive analysis of existing literature, this review seeks to elucidate patterns in the management of PCAAs, evaluate treatment efficacy, and provide insights for improving clinical practice and patient care [28–30].

## Methodology

This systematic review and meta-analysis were conducted in accordance with the Preferred Reporting Items for Systematic Reviews and Meta-Analyses (PRISMA) guidelines. The study aimed to analyze the anatomical variations, clinical presentations, and outcomes associated with pericallosal artery aneurysms by synthesizing the available literature.

### Search strategy

**Fig. 1:**
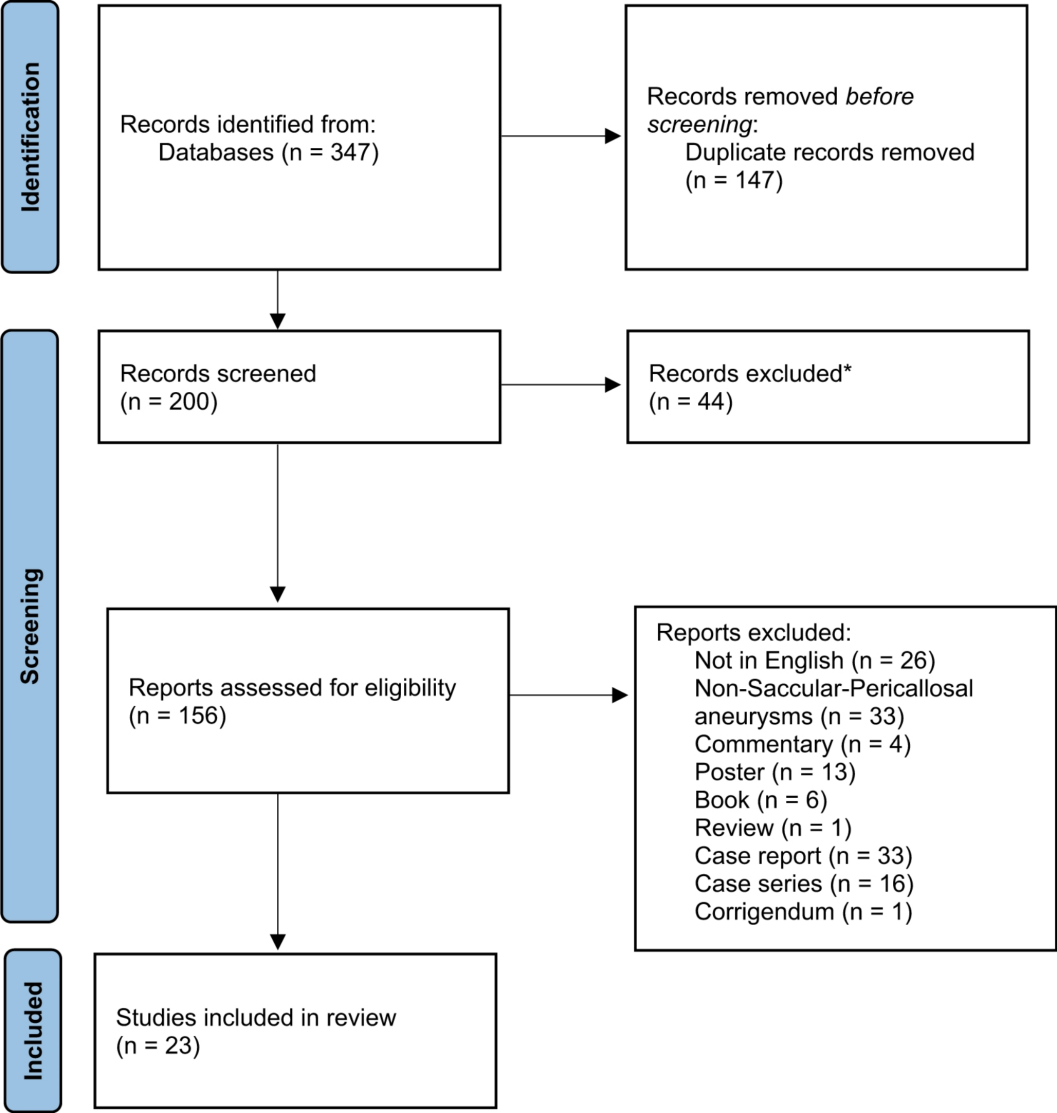
Flow-diagram of the related articles

A comprehensive literature search was conducted across PubMed, ScienceDirect, Scopus, and Google Scholar databases from their inception until June 2024. The search was restricted to studies involving human subjects and published in English. Keywords such as “Pericallosal artery,” “pericallosal artery aneurysm,” and “distal anterior cerebral artery aneurysms” were employed in various combinations to identify relevant articles. The inclusion criteria were strictly limited to human studies published in English that provided full-text access. Exclusion criteria included non-English articles, papers without full-text availability, as well as abstracts, letters to the editor, videos, non-pericallosal aneurysms, and books, to enhance the validity and reliability of the research findings (Fig. 1).

### Study selection

Study selection involved an initial independent screening of titles and abstracts by two authors based on predefined inclusion criteria. Full-text articles were reviewed to verify eligibility. Any discrepancies between authors were resolved through discussion, and a third author was consulted to reach a consensus when necessary. The PRISMA flow diagram was employed to transparently document and illustrate the study selection process, ensuring comprehensive reporting and reproducibility.

### Data extraction

Data extraction was carried out by two independent authors using a standardized form designed to capture critical study attributes, including study design, sample size, patient demographics, anatomical findings, clinical manifestations, and treatment outcomes. To ensure accuracy and consistency, any discrepancies in data extraction were resolved through discussion, with a third author involved as needed. This rigorous approach facilitated a thorough and reliable synthesis of the data for subsequent analysis.

### Outcomes

The outcomes of interest were categorized into anatomical findings, clinical manifestations, and treatment outcomes.

Anatomical Findings: Data were extracted on aneurysm size, rupture status, multiplicity, and associated radiological features such as subarachnoid hemorrhage (SAH), intracerebral hemorrhage, intraventricular hemorrhage, subdural hematoma, and hydrocephalus.

Clinical Manifestations: Information was collected on presenting symptoms, including headache, seizures, and neurological deficits. Where available, preoperative neurological status was assessed using the Glasgow Coma Scale (GCS), and functional status was assessed using the modified Rankin Scale (mRS).

Treatment Outcomes: Postoperative neurological recovery, complications, mortality, and functional status were extracted. When available, changes in mRS scores from preoperative to postoperative states were documented to assess clinical improvement or deterioration.

### Data analysis

The extracted data were analyzed using Python, employing libraries such as Pandas for data manipulation and Matplotlib for data visualization. Descriptive statistics were generated to summarize patient demographics, treatment specifics, and complication rates. Crosstabulations were utilized to explore the association between treatment modalities (microsurgical, endovascular, and combined) and reported complications. Each analysis was tailored to understand the prevalence and impact of various treatment outcomes and to identify patterns within the reported data. Visualizations were created to illustrate the distribution and frequency of treatment-associated complications and outcomes, facilitating a clearer understanding of the data patterns and aiding in the presentation of findings.

### Complications assessment

It focused on vasospasms, intraprocedural rupture, hydrocephalus, bleeding, and other adverse events. Complication incidence was evaluated across microsurgical, endovascular, and combined treatments, with subgroup analyses comparing rates by intervention. Mortality was assessed as an aggregate outcome due to inconsistent subgroup reporting, potentially obscuring the higher risks of ruptured aneurysms.

### Quality assessment

The quality of the included studies was evaluated using the Newcastle-Ottawa Scale (NOS) for cohort and case-control studies and the Joanna Briggs Institute (JBI) critical appraisal checklist for case reports and case series. Studies were classified as high, moderate, or low quality based on their assessment scores, and none of the articles were excluded after the evaluation.

### Selection bias

Most studies clearly defined inclusion criteria and patient selection processes, minimizing selection bias. However, one study (Sun GQ, 2019) lacked detailed descriptions of excluded cases, which may affect generalizability.

### Performance bias

Variability in treatment modalities across centers and potential differences in operator expertise contributed to moderate performance bias.

### Detection bias

The use of advanced imaging techniques, such as MRI and CT angiography, reduced detection bias in all studies.

### Attrition bias

Follow-up data were incomplete in 3 Studies, leading to potential attrition bias. The mean follow-up duration across studies was 20.77 months.

## Results

### Number of studies and patients

Our review analyzed data from 23 studies involving a total of 762 patients. This cohort included both ruptured and unruptured pericallosal artery aneurysms, providing a robust dataset for assessing demographic patterns, risk factors, treatment modalities, and complications.

### Demographics and aneurysm characteristics

**Fig. 2:**
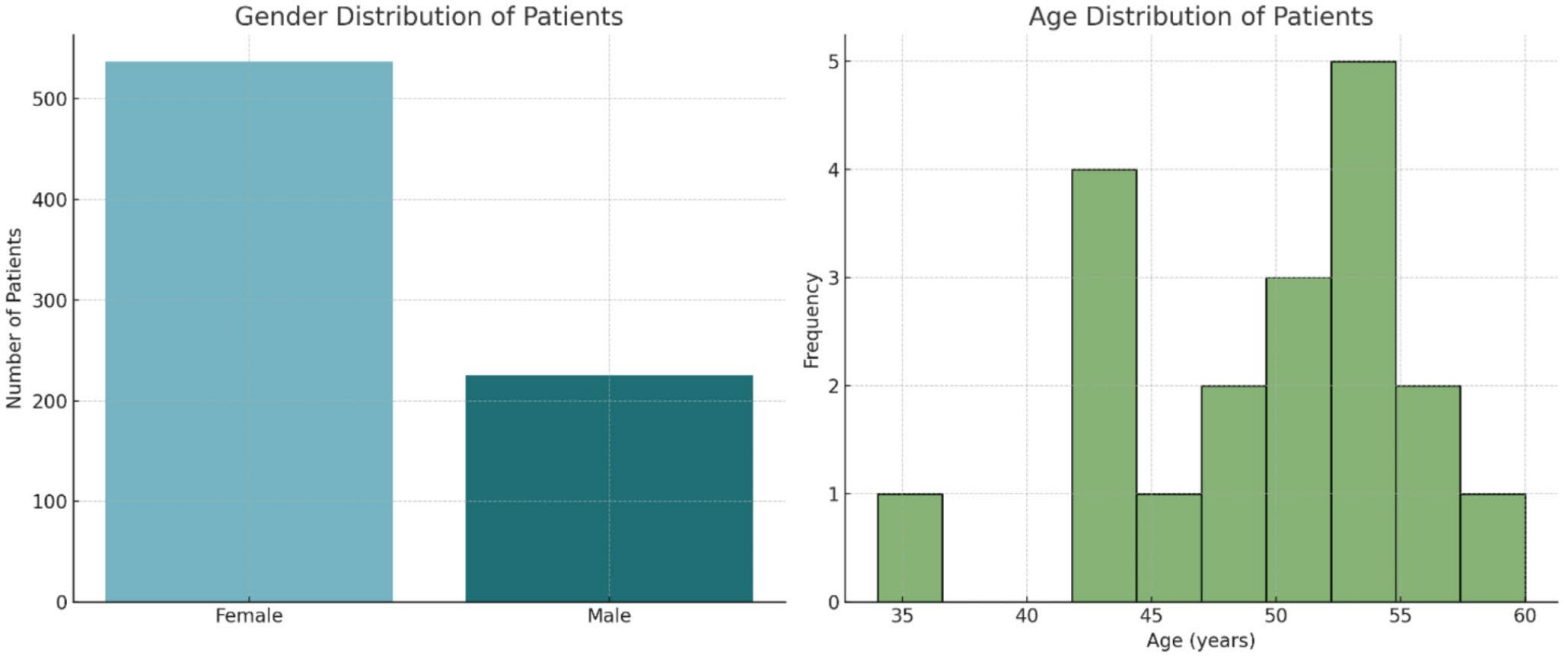
Gender and Age Distribution of Patients. The bar chart illustrates the significantly higher number of female patients compared to male patients. The histogram shows how the ages of the patients are distributed, highlighting a concentration around the median age of 52 years

Demographic analysis of the analyzed studies revealed a notable gender disparity among patients, with females constituting 70.47% (*n* = 537) and males 29.53% (*n* = 225) of the study population (Table 1a). The age distribution of the cohort ranged from 34 to 60 years, with a mean age of 49.93 years (SD = 6.07) (Fig. 2). The examination of patient demographics suggests a higher prevalence of these aneurysms in females and a middle-aged to older adult population.

**Table 1:** a: Demographic characteristics of patients with Pericallosal artery aneurysms

Description	Value
Total Female Patients	537 (70.47%)
Total Male Patients	225 (29.53%)
Mean Age (years)	49.93 ± 6.07
Median Age (years)	52
Minimum Age (years)	34
Maximum Age (years)	60

**Table 1:** b: Detailed characteristics of Pericallosal artery aneurysms

Description	Value
Average Size of Aneurysms (mm)	6.34 ± 3.10
Minimum Size of Aneurysms (mm)	3.3
Maximum Size of Aneurysms (mm)	16.0
Total Number of Ruptured Aneurysms	542
Total Number of Unruptured Aneurysms	251
Studies Reporting Multiple Aneurysms	12

**Table 1:** c: Number of Studies Reporting Associated Radiological Features

Radiological Features	Number of Studies
Hydrocephalus	4 (17.39%)
Intraventricular Hemorrhage	5 (21.73%)
Intracerebral Hemorrhage	7 (30.43%)
Subarachnoid Hemorrhage	20 (86.96%)
Subdural Hematoma	7 (30.43%)

**Fig. 3:**
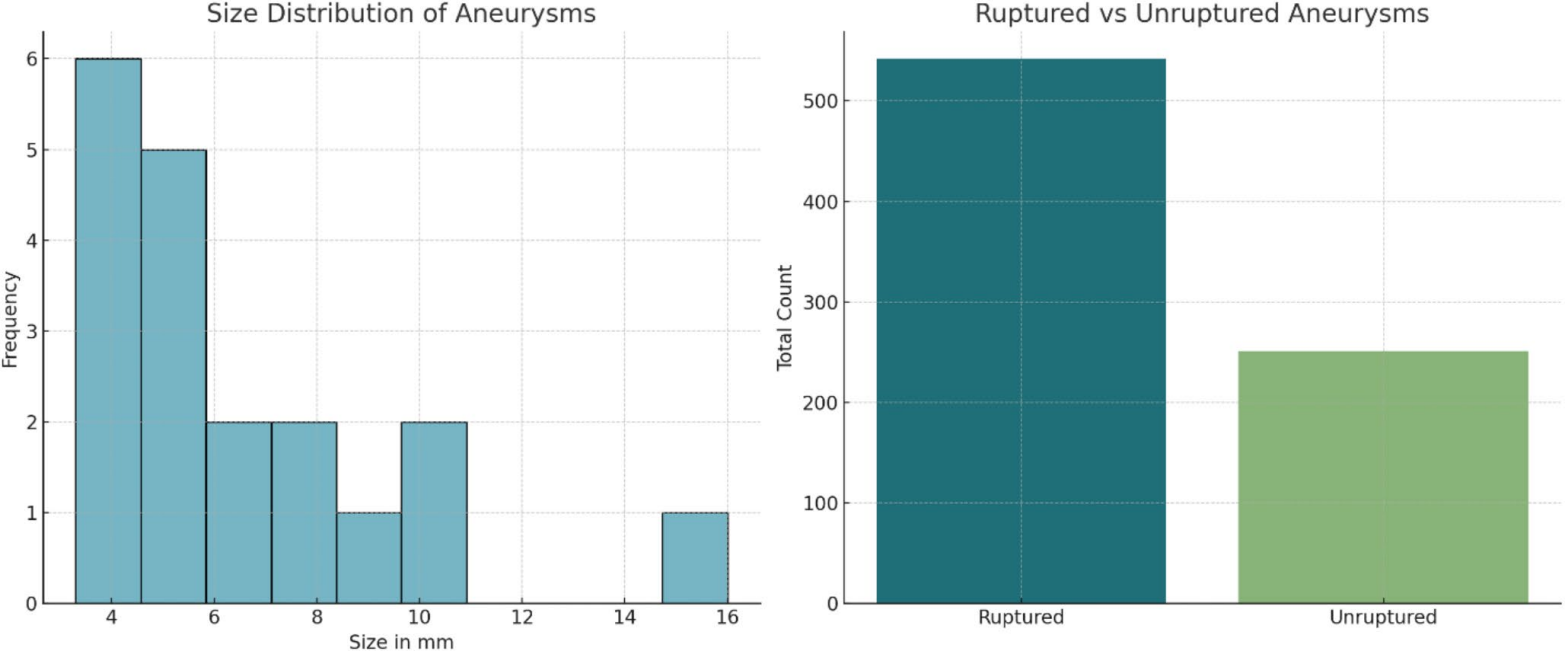
A Histogram of Size Distribution of Aneurysms and Ruptured vs. Unruptured Aneurysms Barchart. The histogram displays the range and frequency of aneurysm sizes within the dataset. The bar chart compares the total counts of ruptured and unruptured aneurysms in the analyzed studies

The average size of aneurysms in the analyzed studies was found to be 6.34 mm with a standard deviation of 3.10 mm. The minimum and maximum sizes recorded were 3.3 mm and 16.0 mm, respectively (Table 1b). There were a total of 542 ruptured and 251 unruptured aneurysms reported across the studies. Notably, in 12 studies, multiple aneurysms were observed among the patients (Fig. 3).

### Risk factors and radiological features

**Fig. 4:**
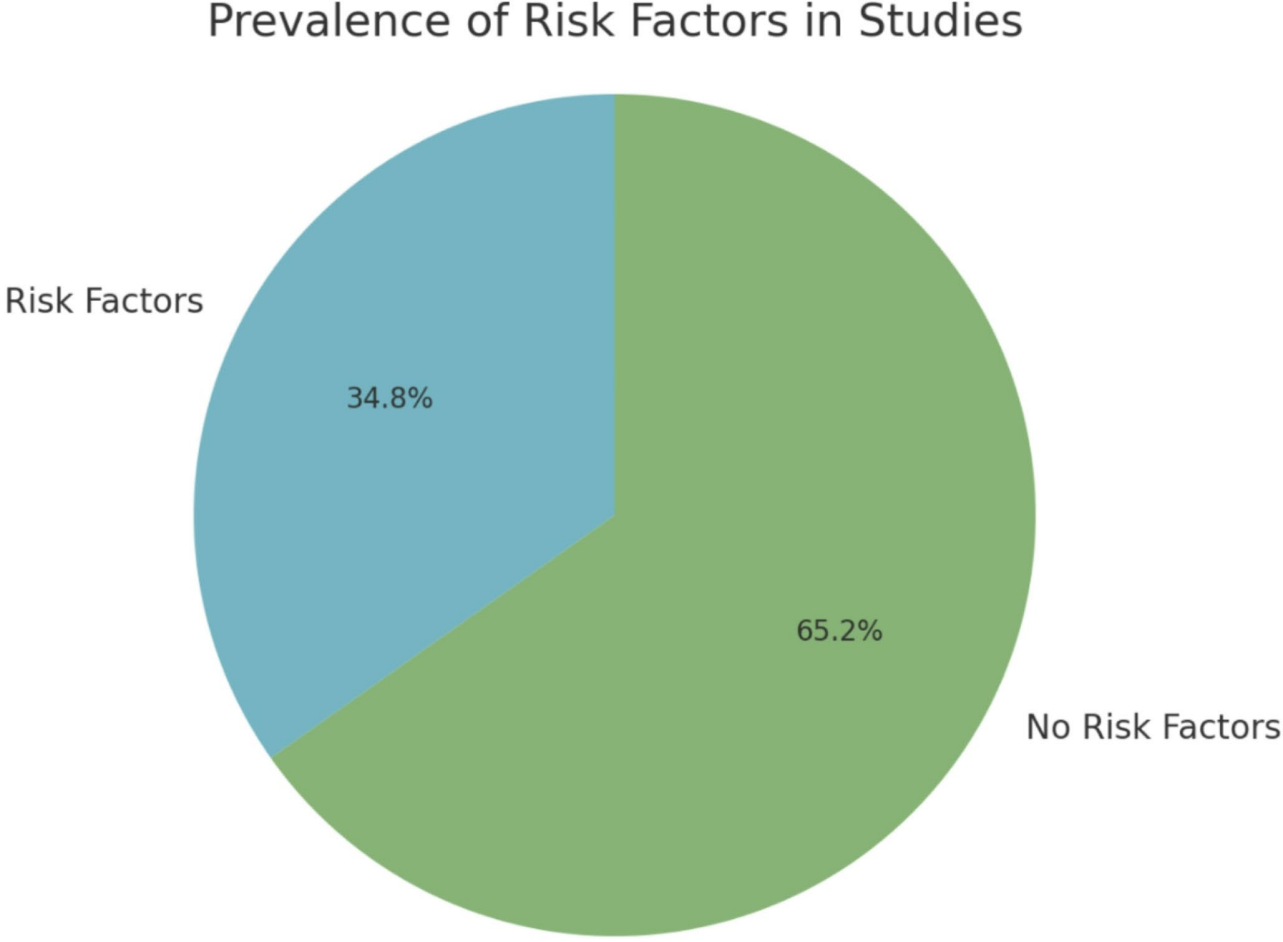
A pie chart of Risk Factors. Illustrating the distribution of studies reporting risk factors relative to those that do not

Of the 23 analyze studies, 8 studies, representing 34.78% of the total, identified specific risk factors associated with the condition (Fig. 4). Among those who had risk factors, smoking was the most prevalent (17.39%). This quantification provides a foundational understanding of how frequently risk factors are reported in the scientific literature concerning pericallosal artery aneurysms.

**Fig. 5:**
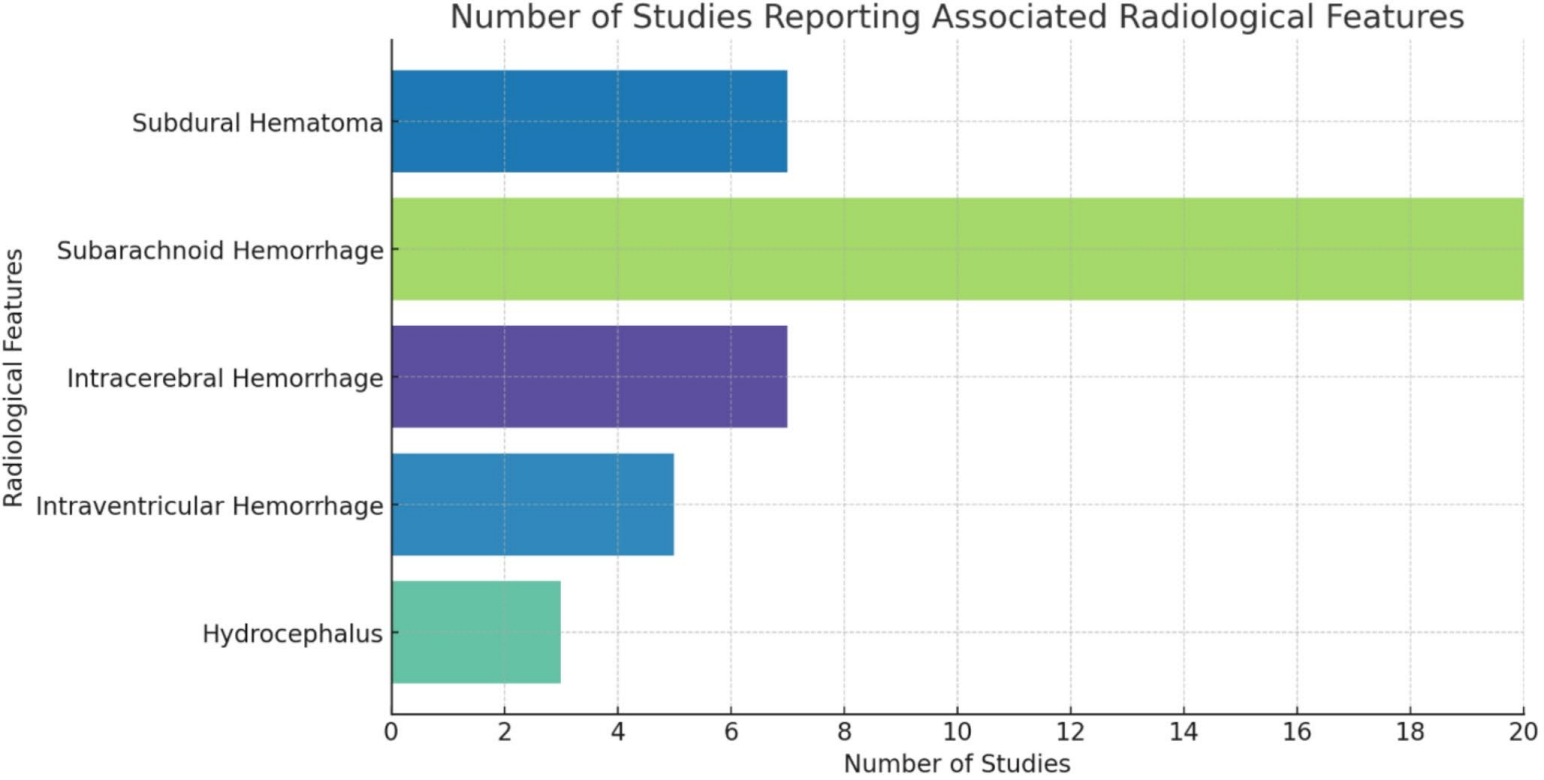
Prevalence of various associated radiological features. A bar chart illustrates the prevalence of various associated radiological features in the analyzed studies, highlighting particularly the higher incidence of subarachnoid hemorrhage

The prevalence of associated radiological features was examined across multiple studies. SAH was reported in 86.96% of studies, making it the most frequently observed pathology (Table 1c). Intracerebral hemorrhage and subdural hematoma were each noted in 30.43% of studies (Fig. 5). Intraventricular hemorrhage was documented in 21.73% of studies, and hydrocephalus in 17.39% of studies.

### Treatment modalities and approaches

**Fig. 6:**
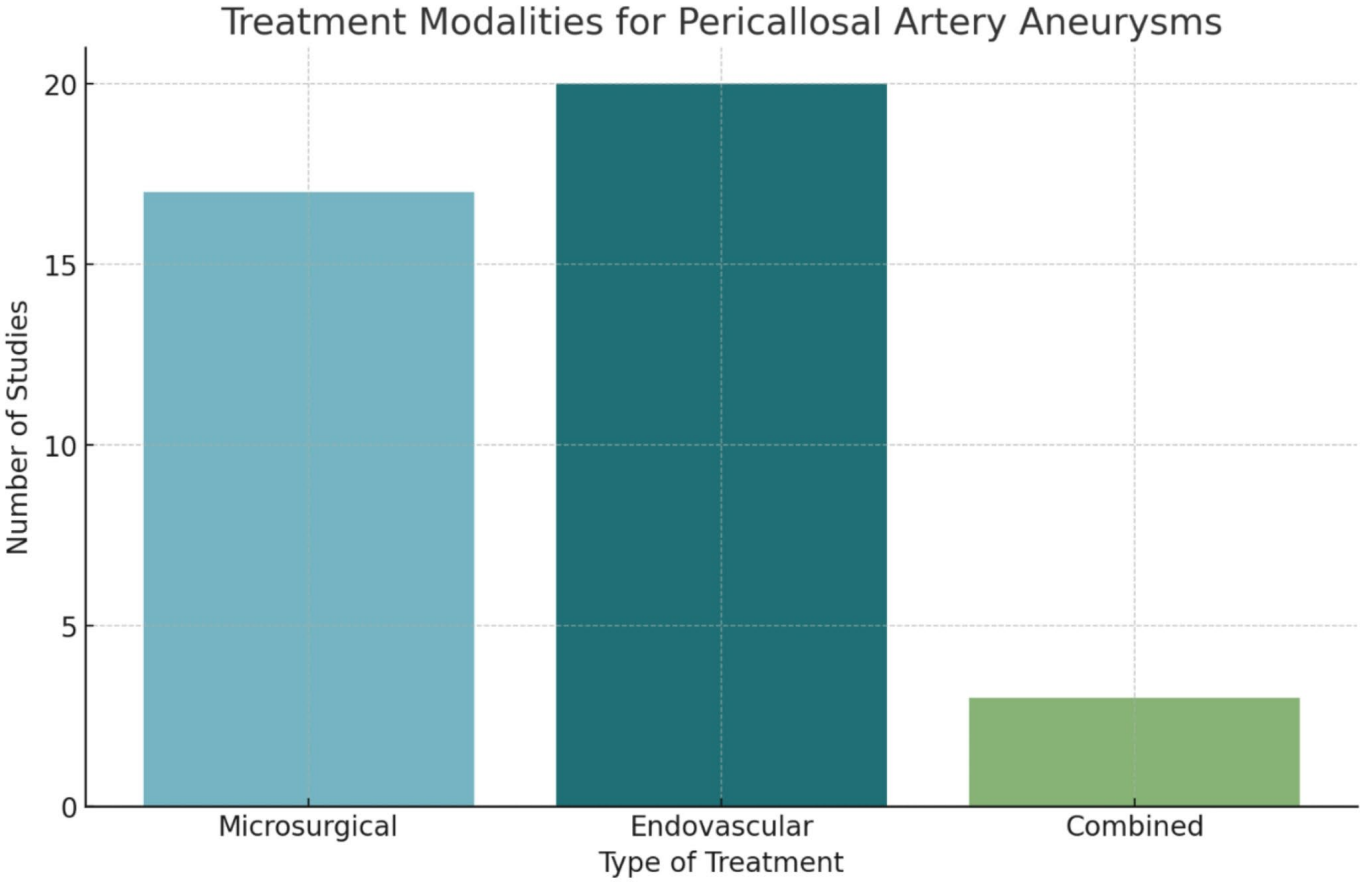
Treatment Modalities. The bar chart illustrates the frequency of each treatment modality reported in studies of pericallosal artery aneurysms

Endovascular treatment was reported in 86.96% of studies, slightly more frequently than microsurgical treatment, which was reported in 73.91%. Combined treatment approaches were noted in 13.04% of studies and those combined treatments mentioned in the studies were not as a result of recurrence (Fig. 6). These percentages exceed 100% because some studies reported using both modalities. Regarding surgical approaches, the majority of studies (60.87%) did not specify the approach used (Table 2a). The interhemispheric approach was the most detailed, reported in 17.39% of studies, while other specific approaches were mentioned only once. It is important to note that not all studies provided detailed counts of procedures, which led us to report the frequency of studies utilizing each treatment approach rather than exact numbers of procedures performed.

**Table 2:** a: Treatment modalities and surgical approaches

Treatment Modality/Surgical Approach		Number of Studies
Treatment Modality	Microsurgical	17 (73.91%)
	Endovascular	20 (86.96%)
	Combined	3 (13.04%)

### Complications and outcome measures

**Fig. 7:**
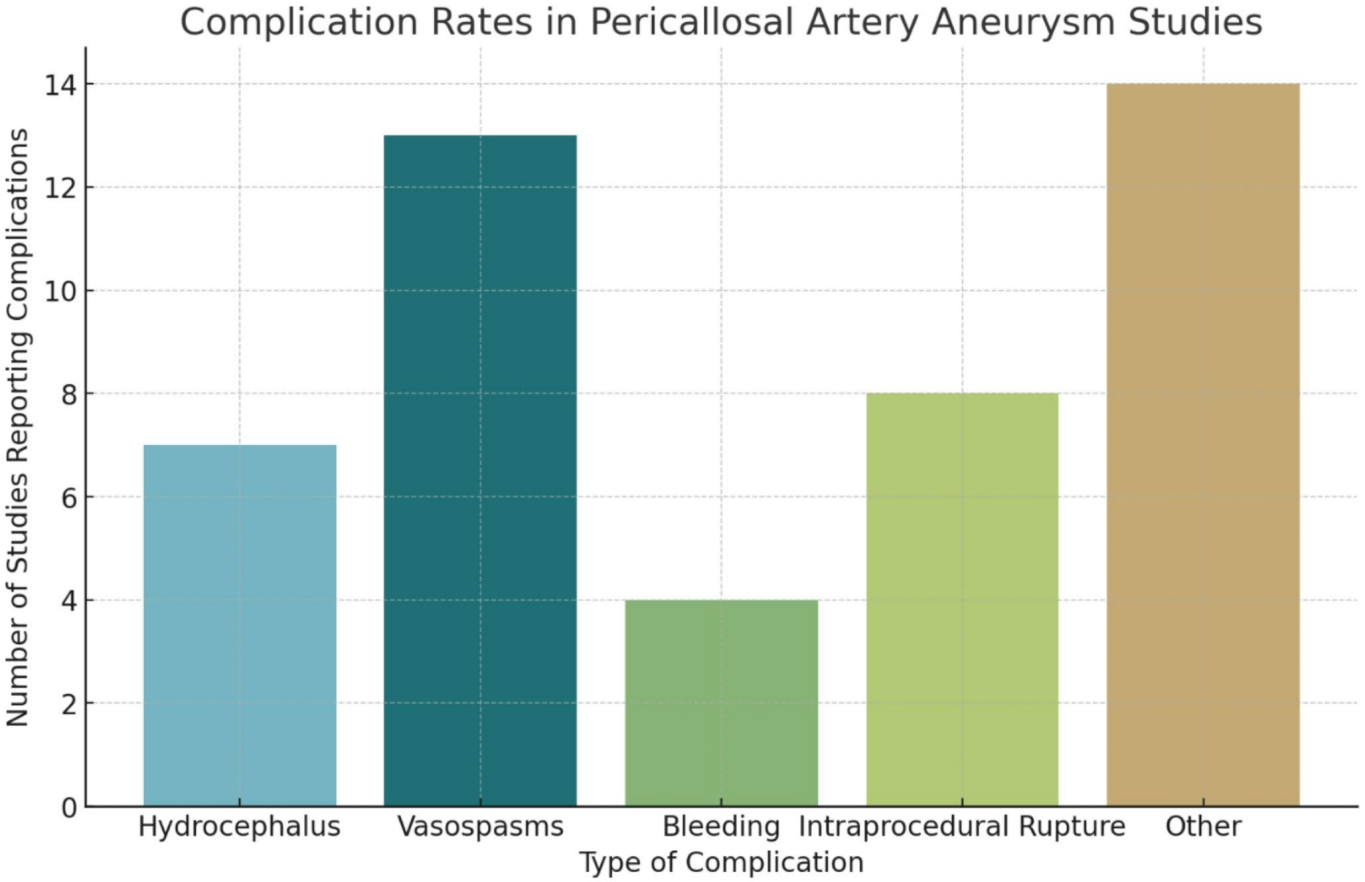
Complication rates. The bar chart illustrates the frequency of each type of complication reported in studies of pericallosal artery aneurysms

Various complications were reported across the included studies. Vasospasms were the most frequently reported complication, noted in 56.5% of studies (Fig. 7). Other complications were documented in 60.9% of studies, highlighting a range of less common issues. Intraprocedural rupture was reported in 34.8% of studies, hydrocephalus in 30.4%, and bleeding in 17.4% studies (Table 2b).

**Table 2:** b: Complication rates in Pericallosal artery aneurysm studies

Reported Complication Type	Number of Studies
Hydrocephalus	7 (30.4%)
Vasospasms	13 (56.5%)
Bleeding	4 (17.4%)
Intraprocedural Rupture	8 (34.8%)
Other Complications	14 (60.9%)

**Fig. 8:**
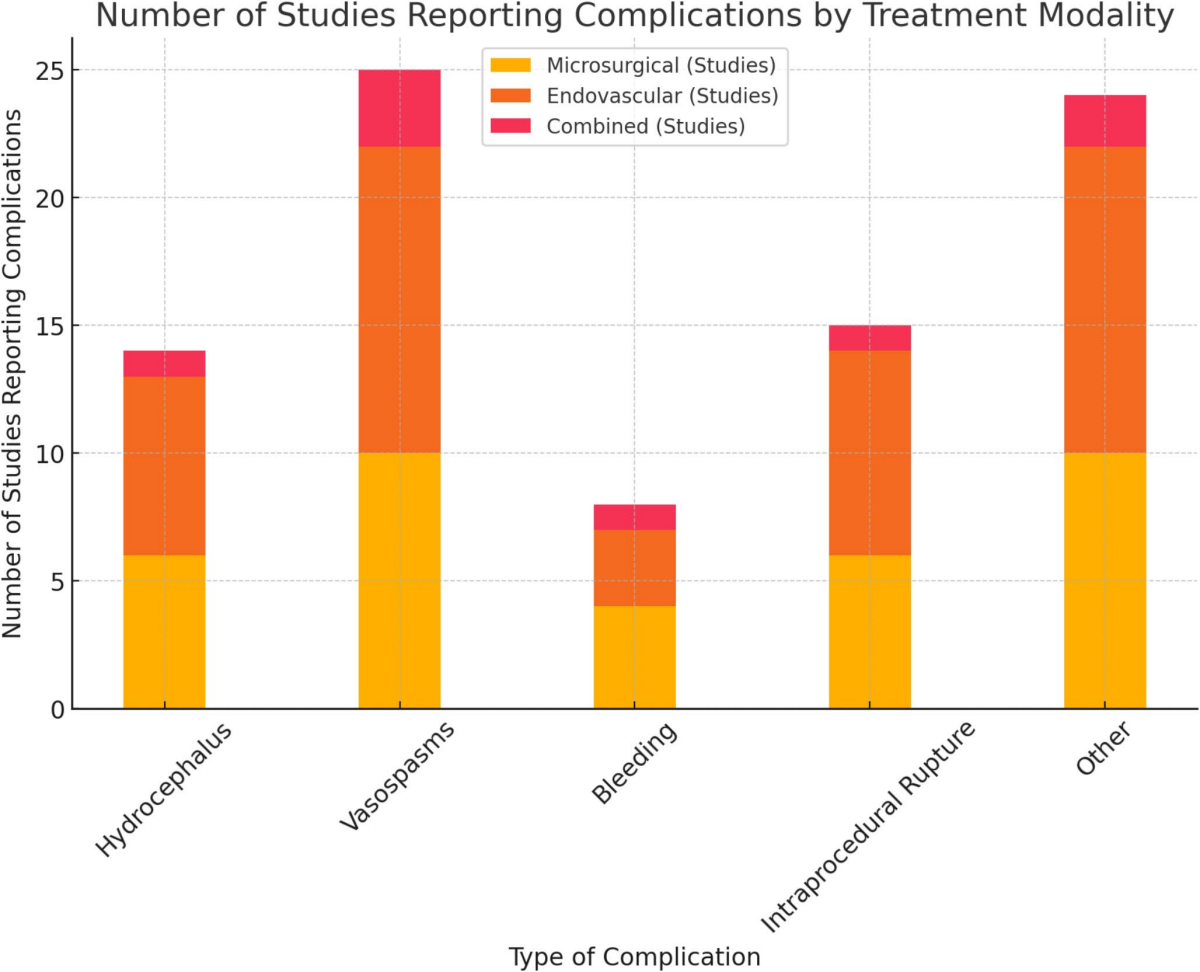
Stacked bar chart depicting the distribution of studies reporting each type of complication for each treatment modality

The association between specific treatment modalities and reported complications was analyzed across the included studies. Hydrocephalus was reported in 26.1% of studies using microsurgical treatments, 30.4% of studies using endovascular treatments, and 4.35% using combined treatments (Table 2c). Vasospasms were noted in 43.5% of studies for microsurgical, 52.2% for endovascular, and 13.04% for combined treatments. Bleeding complications were reported in 17.4% of studies for microsurgical and13.04% for endovascular treatments, with only 1 study reporting it for combined treatments. Intraprocedural rupture was observed in 26.1% of studies for microsurgical, 34.8% for endovascular, and 4.35% for combined treatments. Other complications were mentioned in 43.5% studies using microsurgical treatments, 52.2% using endovascular treatments, and 8.70% using combined treatments (Fig. 8).

**Table 2:** c: Number of studies reporting complications by treatment modality

Complication Type	Microsurgical (Studies)	Endovascular (Studies)	Combined (Studies)
Hydrocephalus	6 (26.1%)	7 (30.4%)	1 (4.35%)
Vasospasms	10 (43.5%)	12 (52.2%)	3 (13.04%)
Bleeding	4 (17.4%)	3 (13.04%)	1 (4.35%)
Intraprocedural Rupture	6 (26.1%)	8 (34.8%)	1 (4.35%)
Other Complications	10 (43.5%)	12 (52.2%)	2 (8.70%)

In our review, the reported overall mortality rate of 6.52% (52 cases) includes outcomes from both ruptured and unruptured pericallosal artery aneurysms. It is important to note that this figure aggregates data from various studies, where specific breakdowns between ruptured and unruptured aneurysms were not consistently reported. This aggregation might mask the typically higher risks associated with ruptured aneurysms and could influence the interpretation of mortality rates.

The mean follow-up duration across studies was 20.77 months, with a standard deviation of 17.19 months, showing variability in the monitoring periods.

## Discussion

Our comprehensive review of PCAA uniquely contributes to the literature by systematically delineating treatment outcomes across endovascular and microsurgical modalities, providing an understanding of their efficacy and associated complications in a particularly challenging subset of cerebral aneurysms.

### Anatomy and embryology of the pericallosal artery (PCA)

PC, originating from the anterior cerebral artery (ACA), is crucial for supplying the corpus callosum and adjacent brain regions [4, 5]. It ascends through the lamina terminalis cistern, traverses the interhemispheric fissure, and enters the callosal cistern above the corpus callosum, following its superior aspect [6]. According to Fischer’s classification, the ACA is divided into several segments: the A2 segment extends from the anterior communicating artery (ACoA) to the junction of the rostrum and genu of the corpus callosum; the A3 segment curves around the genu; and the A4 and A5 segments are located above the corpus callosum [6]. The A3 segment, distal to the calloso-marginal artery (CMA) origin, is often referred to as the PC artery due to its extension into the pericallosal sulcus [31]. The cingulate gyrus differentiates the CMA from the PCA, though the term “pericallosal artery” is frequently misapplied to the A2 segment [5, 6, 32].

Anatomical variations include the bi-hemispheric ACA, where one side terminates in the cingulate sulcus and is supplemented by a collateral branch from the contralateral PCA. This branch crosses the midline through a foramen in the falx cerebri, supplying the majority of the PCAA territory on the opposite side [6]. The artery’s termination remains debated; it may either persist in its fetal form to supply the splenium or regress, with splenium blood supply provided by branches of the posterior cerebral artery [4]. Understanding these variations is crucial for neurosurgical and endovascular procedures to avoid neurological deficits [5, 16, 17].

The development of the corpus callosum and its vascularization occurs between the 10th and 12th weeks of gestation. Vascularization is detectable in 85% of cases between the 12th and 21st weeks using high-definition Doppler imaging, originating from the ACA [33, 34]. By 11 weeks, the artery ascends in the lamina terminalis cistern, travels through the interhemispheric fissure, and enters the callosal cistern above the corpus callosum. As gestation progresses, the artery follows the superior aspect of the corpus callosum, becoming more defined, with its main branches visible by 16 weeks [33]. This developmental process reflects its essential role in supplying the growing brain.

### Aneurysms of the pericallosal artery

#### Prevalence and risk factors

PCAAs are relatively rare, comprising less than 9% of all intracranial aneurysms and about 4% of ruptured cases [1–3, 35]. Genetic and hormonal factors are significant in their occurrence, with a higher prevalence noted in females and individuals aged 40 to 60 years [7–12]. Estrogen’s role is particularly noted in this gender disparity [7, 8, 11]. While congenital causes of PCAAs exist, those in atypical locations have also been linked to vascular anomalies, trauma, and infections [3]. PCAAs multiplicity is not uncommon, with some studies reporting rates over 40% [3, 36], and there are documented cases of iatrogenic PCAAs [15, 37]. The fact that over a third of studies identified risk factors highlights their significant role in PCAAs development, confirming the influence of genetic, physiological, or lifestyle factors on their pathogenesis [13, 14]. Our demographic analysis reveals a significant gender disparity with females constituting 70.47% of the study population and males 29.53%. The age distribution of patients ranged from 34 to 60 years, with a mean age of 49.93 years.

### Clinical and radiological features

Unruptured PCAAs can present with clinical variability that may lead to misdiagnosis. Symptoms such as sudden, severe headaches, vomiting, transient loss of consciousness, neck pain, nuchal stiffness, and incontinence can mimic those of SAH [15]. For instance, a patient with systemic hypertension might experience these symptoms, which can also include personality changes, complicating diagnosis [15–17]. Additionally, aneurysms may be mistakenly identified as space-occupying lesions like meningiomas, resulting in symptoms such as headaches, seizures, and speech alterations [18]. Advanced imaging techniques, including CT scans, MRIs, and digital cerebral angiography, are crucial for accurate diagnosis, revealing the aneurysm’s true nature and its potential mass effect on adjacent brain structures (18).

### Demographics and radiological findings

Cerebral aneurysms typically measure between 5 and 7 mm in diameter, though they can range from 2 mm to 25 mm [38]. Larger aneurysms generally have a higher rupture risk and more severe clinical implications [39]. Research shows that both small (< 5 mm) and large (≥ 5 mm) ruptured intracranial aneurysms exhibit higher Size Ratio (SR), Undulation Index (UI), Ellipticity Index (EI), and Oscillatory Shear Index (OSI), and lower Wall Shear Stress (WSS) compared to unruptured ones. Small ruptured aneurysms are more common in patients with multiple aneurysms, while large ones are associated with higher maximum WSS and younger patients [39]. Similar patterns in morphological parameters, such as increased aspect ratios and inflow angles, have been observed [9].

Despite their smaller size, PCAAs carry a high risk of rupture, leading to significant morbidity and mortality if untreated [36, 37]. Our study found an average aneurysm size of 6.34 mm, with a range from 3.3 mm to 16.0 mm, indicating that PCAAs often fall within the smaller range of aneurysms. This variability in size, coupled with the associated clinical risks, suggests that rupture risk is influenced by both aneurysm size and other factors. For example, even small aneurysms in the posterior communicating artery (Pcom), with diameters less than 4 mm, can have a significant rupture risk [40].

Aneurysm location and morphology further complicate treatment. For instance, aneurysms at the junction of the pericallosal and callosomarginal arteries, as well as those in the anterior communicating artery (ACom), pose additional challenges for both surgical and endovascular approaches [41–43]. Most aneurysms are saccular (berry), characterized by a round sac with a narrow neck, though fusiform aneurysms also occur [44].

Distal anterior cerebral artery (a3) aneurysms, including PCAAs, often rupture at smaller sizes compared to other intracranial aneurysms, indicating a need for proactive treatment even in small, unruptured cases [45]. Effective surgical intervention requires careful dissection, with outcomes ranging from full recovery to severe disability or death [45]. Our study highlights a higher incidence of ruptured PCAAs, suggesting these aneurysms might have subtle or asymptomatic features that predispose them to rupture or result in delayed diagnosis [8–10, 27]. The frequent reporting of SAH underscores its critical role that impacts the clinical course of aneurysms, stressing the need for vigilant management and individualized treatment plans considering patient age, risk factors, and aneurysm characteristics, as rupture risk does not always correlate with aneurysm size across different cerebral arteries [46].

### Treatment modalities and approaches

**Fig. 9:**
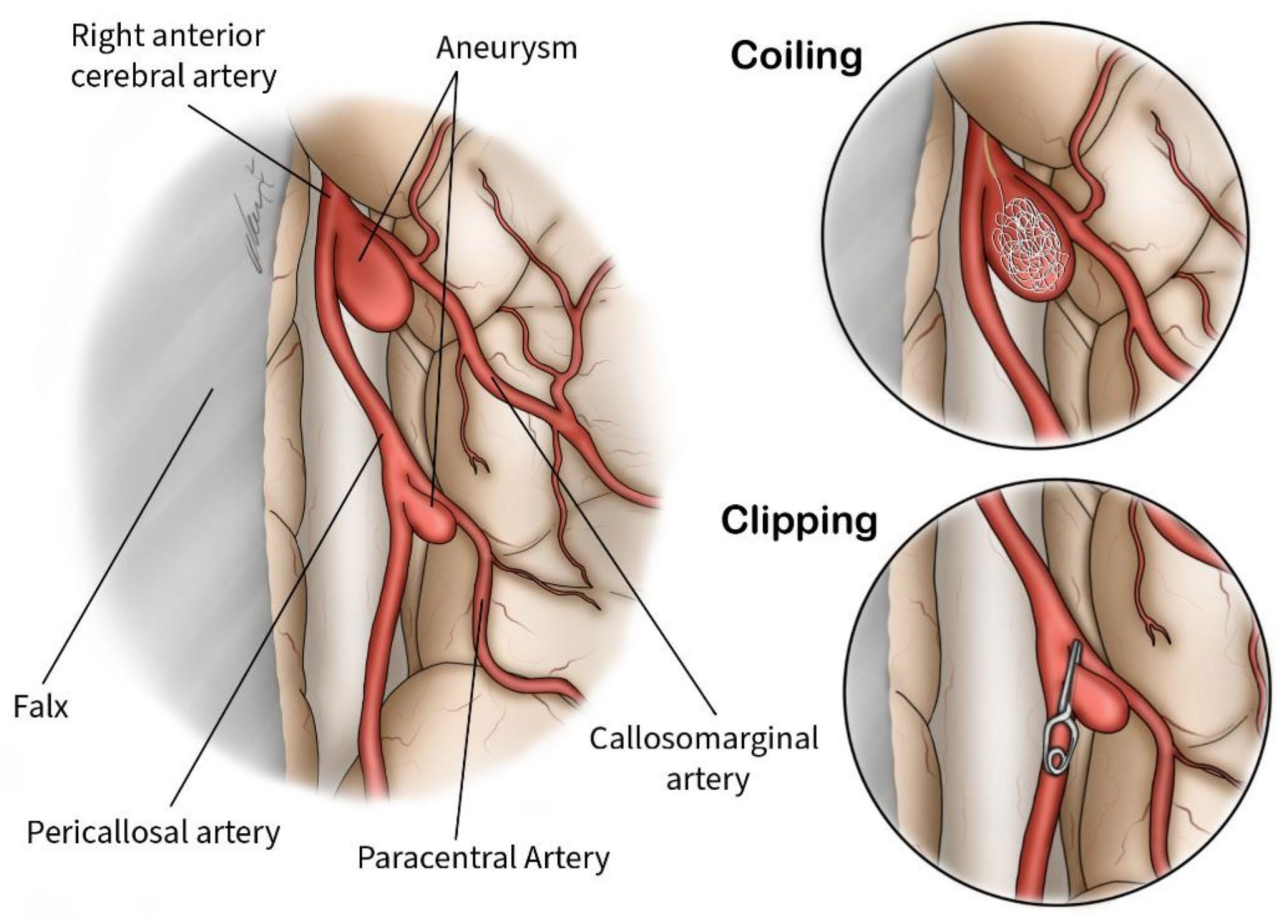
An anatomical illustration showing the treatment modalities of Pericallosal artery aneurysm

Evidence from retrospective studies demonstrates that both microsurgical and endovascular approaches are technically viable and generally result in favorable outcomes for managing pericallosal artery aneurysms (Fig. 9) [2]. The trend toward endovascular treatments has grown due to their minimally invasive nature, bolstered by technological advancements and improved patient outcomes [10]. Among these techniques, coil embolization is commonly used for its effectiveness and minimally invasive approach, leading to high success rates [47]. Flow diverters are effective for large or complex aneurysms by redirecting blood flow to induce thrombosis and closure [21, 22]. Onyx liquid embolization is employed for aneurysms with irregular shapes or wide necks, where the embolic agent solidifies upon contact with blood to occlude the aneurysm [23]. Stent-assisted coiling, which involves placing coils with a stent, helps stabilize the coils and improve outcomes for wide-necked aneurysms [48, 49]. In cases of challenging anatomy, such as tortuous vessels or SAH, balloon inflation in the proximal middle cerebral artery can facilitate access to the A1 segment of the anterior cerebral artery by creating a rebound effect [24]. In a study by Lv et al. (2020), the Pipeline flow-diverting stent was shown to be a viable treatment option for complex cerebral aneurysms, demonstrating high occlusion rates with minimal complications over a follow-up period of 6 to 12 months. These findings support the use of flow-diverting stents [50]. The choice of technique is tailored to the aneurysm’s characteristics and the patient’s condition to ensure optimal results with minimal invasiveness. In our study endovascular treatment was reported in 86.96% of studies.

Surgical clipping approaches vary depending on the aneurysm’s location, size, and complexity. The interhemispheric approach is commonly used, offering direct access to midline aneurysms with minimal brain manipulation [51–53]. For ruptured aneurysms with high rupture risk, combining the anterior interhemispheric approach with the pterional or subfrontal approach can facilitate early proximal vascular control [20]. The pterional approach, involving craniotomy near the pterion, provides a broad view and can be combined with other techniques for enhanced control [54]. The median supraorbital keyhole approach is effective for clipping.

distal anterior cerebral artery aneurysms (DACA) aneurysms and is also used for pericallosal and frontopolar artery aneurysms, offering good access, control, and cosmetic results [55]. In our study, surgical management was reported in 86.96% of the included studies. Among these, 17.39% specifically utilized the interhemispheric approach, while the remaining studies did not specify the surgical technique used. The limited detail on surgical approaches in many reports highlights the need for standardized documentation. Improved reporting will enhance research comparability and reproducibility.

In the management of PCAAs, various bypass techniques have been utilized, each with different outcomes. An A2-A3 fusiform aneurysm can be addressed through a radial artery graft bypass with proximal occlusion or by reimplanting the callosomarginal artery into the pericallosal artery with distal occlusion. Both methods can achieve aneurysm obliteration [28, 56]. For A3 aneurysms, treatment options were documented like segment with aneurysm excision and reanastomosis or a vertical side-to-side anastomosis between the distal callosomarginal and pericallosal arteries. However, direct reanastomosis has been associated with bypass occlusion [56, 57]. No bypass techniques were documented in the retrospective analyses included in our systematic review.

#### Complications and outcome measures

The primary complication of PCAAs is rupture, often leading to significant intracerebral hemorrhage and hematoma formation, typically in the frontal lobe, pericallosal cistern, or cingulate gyrus. This results in elevated intracranial pressure and severe neurological deficits [58]. Our study confirms these findings, with 56.5% of studies reporting vasospasms and 87% documenting SAH. Additionally, intraventricular hemorrhage was noted in 21.73% of studies, and hydrocephalus in 17.39%, underscoring the serious nature of these complications.

The timing of intervention significantly impacts complication rates. Literature suggests that earlier intervention, within 96 h of hemorrhage, results in fewer complications compared to treatment administered 14 days post-bleeding [3]. Our results corroborate this, showing varied complication rates across treatment modalities. Hydrocephalus, for instance, was reported in 26.1% of studies using microsurgical techniques, 30.4% with endovascular treatments, and only 4.35% in studies using combined treatments. This variation highlights how timely intervention and treatment type can influence outcomes.

The prevalence of vasospasms and intraprocedural ruptures illustrates the inherent risks of treating PCAs [25]. Mortality rates for PCAAs highlight the severity of the condition, with a reported mortality rate of 6.52% in our analysis [25, 51, 59]. The integration of mortality rates from both ruptured and unruptured PCAAs in our analysis highlights a significant challenge in synthesizing literature where detailed outcomes based on rupture status are not uniformly reported. Future studies would benefit from a standardized approach to reporting outcomes separately for ruptured and unruptured aneurysms to enable more precise assessments of treatment efficacy and risk. Our finding supports earlier findings that PCAAs, particularly those with severe SAH, are associated with high mortality and morbidity rates [3].

Surgical clipping is associated with several risks, including postoperative neurological deficits, especially when substantial brain retraction or manipulation is necessary. Other risks include surgical site infections, complications related to anesthesia, and intraoperative hemorrhage [60]. Our study also identified complications arising from SAH, such as hydrocephalus, vasospasm, and cerebral ischemia, which require meticulous intensive care management [60]. Specifically, our analysis revealed that vasospasms occurred in 43.5% of cases, bleeding complications were observed in 17.4%, and intraprocedural rupture was noted in 26.1%.

Endovascular management can result in incomplete aneurysm occlusion, which may necessitate further interventions or lead to aneurysm recurrence. Our findings indicate a 13% recurrence rate within six months, with complications such as coil migration or compaction also observed [60]. Mortality associated with SAH was noted in 9.1%, underscoring the severe nature of ruptured aneurysms [60]. Specifically, our study found that vasospasm occurred in 52.2% of cases, bleeding complications were noted in 13.04%, and intraprocedural rupture was present in 34.8% of studies.

Several factors influence the prognosis of PCAAs. The severity of SAH at initial presentation is a critical determinant, with higher grades generally indicating worse outcomes [61]. Intracerebral hematoma also significantly impacts prognosis. Recurrent bleeding before surgical intervention complicates the condition further [30]. Our findings highlight the importance of timely surgical intervention, as well as the impact of factors such as patient age, the presence of multiple aneurysms, and the experience of the surgeon on overall outcomes [29].

### Limitations

This systematic review has several limitations. The variability in reporting across studies, including incomplete details on surgical approaches and treatment modalities, complicates comparisons and limits the reliability of our conclusions. Differences in patient demographics, aneurysm characteristics, and treatment protocols contribute to heterogeneity, affecting the generalizability of the findings. Additionally, the average follow-up duration of 20.77 months varies widely, impacting long-term outcome assessments. Some treatment modalities, such as bypass techniques, were not covered in the literature, and retrospective study designs introduce potential biases. Also, our review’s ability to differentiate between the outcomes of ruptured versus unruptured PCAAs is limited by the inconsistent reporting across the included studies. The overall mortality rate presented, therefore, combines data from these two distinct groups, potentially confounding the true risk profile associated with each. This limitation shows the need for more granular data in future research, which could provide deeper insights into the specific risks and benefits of treatments for ruptured and unruptured PCAAs. Future research should focus on standardized reporting, larger sample sizes, and prospective study designs to address these limitations.

## Conclusion

This systematic review offers a thorough examination of PCAAs and reveals several key insights. A significant gender imbalance was observed, with females representing the majority of cases. The average age of patients was 49.93 years, suggesting a higher incidence among middle-aged individuals. The reviewed studies indicated that PCAAs typically measure 6.34 mm in diameter and are often associated with SAH, reported in nearly 87% of cases. Endovascular treatments were more commonly employed than microsurgical options, yet they were linked to higher complication rates, including vasospasm and hydrocephalus. Microsurgical treatments also carried risks, such as intraprocedural rupture. The overall mortality rate was 6.52%. To enhance the management of PCAAs, future research should aim to standardize treatment protocols and improve the consistency of reporting practices.

## Electronic supplementary material

Below is the link to the electronic supplementary material.

Supplementary Material 1

## Data Availability

No datasets were generated or analysed during the current study.
