# Supplementary material for: Pericallosal artery aneurysms: an evidence-based analysis of clinical presentations, therapeutic approaches, and outcome
Source: Neurosurg Rev. 2025 Apr 3;48(1):348. doi: 10.1007/s10143-025-03500-6 (PMC11965253; doi:10.1007/s10143-025-03500-6)
Supplement: Supplementary file 1 — Supplementary Material 1 [file 10143_2025_3500_MOESM1_ESM.docx]

Abbreviations Table:

| **Abbreviation** | **Full Form** | **Abbreviation** | **Full Form** |
| --- | --- | --- | --- |
| **PCAA** | Pericallosal artery aneurysm | **PCA** | Pericallosal artery |
| **SAH** | Subarachnoid hemorrhage | **CT** | Computed tomography |
| **MRI** | Magnetic resonance imaging | **ACA** | Anterior cerebral artery |
| **ACoA** | Anterior communicating artery | **CMA** | Callosomarginal artery |
| **DACA** | Distal anterior cerebral artery | **Pcom** | Posterior communicating artery |
| **SR** | Size Ratio | **UI** | Undulation Index |
| **EI** | Ellipticity Index | **OSI** | Oscillatory Shear Index |
| **WSS** | Wall Shear Stress |  |  |

**Abbreviations Table: illustrating the used abbreviations in the study**
